# Identifying pre-disease signals before metabolic syndrome in mice by dynamical network biomarkers

**DOI:** 10.1038/s41598-019-45119-w

**Published:** 2019-06-24

**Authors:** Keiichi Koizumi, Makito Oku, Shusaku Hayashi, Akiko Inujima, Naotoshi Shibahara, Luonan Chen, Yoshiko Igarashi, Kazuyuki Tobe, Shigeru Saito, Makoto Kadowaki, Kazuyuki Aihara

**Affiliations:** 1 Division of Kampo Diagnostics, Institute of Natural Medicine, University of Toyama, Toyama, 930-0194 Japan; 2 Division of Chemo-Bioinformatics, Institute of Natural Medicine, University of Toyama, Toyama, 930-0194 Japan; 3 Division of Gastrointestinal Pathophysiology, Institute of Natural Medicine, University of Toyama, Toyama, 930-0194 Japan; 4 CAS Center for Excellence in Molecular Cell Science, Institute of Biochemistry and Cell Biology, Shanghai Institutes for Biological Sciences, Chinese Academy of Sciences, Shanghai, 200031 China; 5 Institute of Industrial Science, The University of Tokyo, Tokyo, 153-8505 Japan; 6 First Department of Internal Medicine, Faculty of Medicine, University of Toyama, Toyama, 930-0194 Japan; 7 Department of Obstetrics and Gynecology, Faculty of Medicine, University of Toyama, Toyama, 930-0194 Japan; 8 International Research Center for Neurointelligence (WPI-IRCN), The University of Tokyo Institutes for Advanced Study, The University of Tokyo, Tokyo, 113-0033 Japan

**Keywords:** Predictive markers, Metabolic syndrome

## Abstract

The establishment of new therapeutic strategies for metabolic syndrome is urgently needed because metabolic syndrome, which is characterized by several disorders, such as hypertension, increases the risk of lifestyle-related diseases. One approach is to focus on the pre-disease state, a state with high susceptibility before the disease onset, which is considered as the best period for preventive treatment. In order to detect the pre-disease state, we recently proposed mathematical theory called the dynamical network biomarker (DNB) theory based on the critical transition paradigm. Here, we investigated time-course gene expression profiles of a mouse model of metabolic syndrome using 64 whole-genome microarrays based on the DNB theory, and showed the detection of a pre-disease state before metabolic syndrome defined by characteristic behavior of 147 DNB genes. The results of our study demonstrating the existence of a notable pre-disease state before metabolic syndrome may help to design novel and effective therapeutic strategies for preventing metabolic syndrome, enabling just-in-time preemptive interventions.

## Introduction

Metabolic syndrome is a state wherein a blood glucose level, blood pressure, and/or a triglyceride level are elevated higher than those in normal ranges, mainly due to abdominal obesity^1^. A reduced level of high-density lipoprotein (HDL) cholesterol is also observed^1^. Metabolic syndrome increases the risk of lifestyle-related diseases, including type 2 diabetes, cardiovascular disease (CVD), and nonalcoholic steatohepatitis (NASH)^1–3^. Diabetic retinopathy is a major cause of blindness in adults. Moreover, lethal heart attacks and strokes are ultimately induced from arteriosclerosis originating from metabolic syndrome^3^. NASH can progress to hepatocellular carcinoma (HCC)^2^. The incidence of metabolic syndrome is increasing because of the lack of effective treatment methods. The incidence of obesity, a major cause of metabolic syndrome, also continues to increase worldwide. According to the World Health Organization (WHO), in 2016, more than 650 million (13%) adults were obese, with body mass index (BMI) ≥30 *kg*/*m*^2^^4^. The number of individuals with obesity has increased nearly three times since 1975^4^. Therefore, there is a need for the development of new therapeutic strategies for metabolic syndrome, particularly in the early disease stage, which is considered as the best period for effective treatment. One approach is to focus on the *pre-disease state*^5^ (that is, the most susceptible state to disease progression) before metabolic syndrome and elucidate the complex mechanisms underlying the transition from a relatively healthy condition or mild obesity to metabolic syndrome. It is important to note that the pre-disease state does not mean the absence of any noticeable changes at any part of the body. Instead, it is the state that is (1) not diagnosed as the disease state based on the existing criteria but (2) supposed to have high susceptibility or vulnerability to diseases.

Recently, we proposed mathematical theory for the detection of the pre-disease state before complex/multifactorial diseases, called the dynamical network biomarker (DNB) theory^5^. The main purpose of this theory is to detect early warning signals of *critical transitions*^6^ in biological systems. Critical transitions are sudden and large-scale state transitions that occur in many complex systems, such as ecological systems^7^, climate systems^8^, financial markets^9^, microorganism populations^10^, and the human body^5^. It is important to note that critical transitions are different from critical phenomena and phase transitions in physics. Recent studies revealed that the early warning signals of critical transitions in various systems share some common features. For example, increases in variance and auto-correlation, and decreases in recovery rates are observed frequently^6^. Furthermore, increases in the strength of intervariable correlations^11^ have been reported. This is not necessarily because the coupling between variables becomes stronger by itself near a critical transition. A possible mechanism is that when variances of variables increase, their inherent interactions may become much apparent, resulting in stronger correlations.

In the context of the DNB theory, a state transition from a healthy state to a disease state is regarded as a critical transition if (1) quick recovery to the healthy state becomes difficult or even impossible once the transition occurs, and (2) intervention and prevention as treatment before the transition, that is, at the pre-disease state or earlier, are much easier than those after the transition. The idea regarding the development of a disease as a critical transition has been widely accepted in the literature^5,12–17^.

The DNB theory provides statistical methods to select relevant variables for detection of the pre-disease state. The basic assumption is that a small number of closely related variables, called DNBs, convey early warning signals for the impending critical transition. The DNB theory and its extensions have also proposed several measures for the detection of the pre-disease state^5,14,16^. For example, the average standard deviation of DNB variables and the average absolute value of correlation coefficients between DNB variables are easy to calculate and are widely applicable measures. Simultaneous changes in these measures are regarded as a reliable early warning signal, suggesting that a critical transition to a disease state will occur. On the other hand, measures based on auto-correlation and recovery rates with respect to DNB variables were not considered in previous studies mainly because these statistics require unrealizable repetitive measurements at short intervals from the same individual.

The DNB theory has been applied to real data of many diseases, such as acute respiratory distress syndrome^5^, diabetes mellitus^12^, influenza^14^, cancer^13–15,17^, and Alzheimer’s disease^16^, as well as experimental data in cell biology, such as that of cell differentiation^18^. Moreover, researches on the improvement of the statistical methods^14,16^ and refinement of the theory^19^ are in progress.

Metabolic syndrome is characterized by several disorders, such as hyperglycemia, hypertension, and dyslipidemia, all of which are caused by the accumulation of visceral fat due to overeating and lack of exercise^1,3^. Therefore, many animal models, such as ob/ob and db/db mice, have been established^20^ in order to elucidate the mechanisms underlying metabolic syndrome and construct new therapeutic strategies. Tsumura, Suzuki, Obese, Diabetes (TSOD) mice^21,22^ are known to develop a wide range of disorders similar to human metabolic syndrome, including hyperglycemia^22^, hypertension^23^, dyslipidemia^22^, glucose intolerance^21^, insulin resistance^24^, peripheral neuropathy^25^, intestinal dysbiosis^26^, and histopathological characteristics in the liver similar to those of human nonalcoholic fatty liver disease (NAFLD)^27^.

In the present study, we used the DNB theory to detect the pre-disease state, which is known as *Mibyou*^28^ in traditional Japanese medicine and *Wei Bing*^29^ in traditional Chinese medicine, before metabolic syndrome in TSOD mice.

## Results

**Figure 1:**
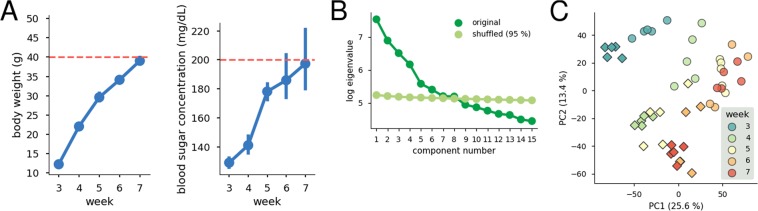
Body weights, blood sugar concentrations, and PCA of the transcriptome data. (**A**) Body weights and blood sugar concentrations measured from TSOD mice. Error bars show 95% confidence intervals. Red dashed lines show the suggested thresholds for defining obesity in TSOD mice (body weight ≥40 g) and prediabetes in rodents (blood glucose level ≥200 mg/dL). (**B**) Scree plot of the largest 15 components. The number of meaningful PCs is estimated to be the number of eigenvalues calculated from the original transcriptome data larger than the 95 percentiles of eigenvalues calculated from shuffled data. For more details, see *Dimensionality reduction* in Materials and Methods. (**C**) PCA plot of the transcriptome of TSOD mice (circles) and TSNO mice (diamonds). The numbers in parentheses denote explained variance ratios corresponding to each PC. Regarding results of other meaningful components, see Supplementary Fig. S1. PC: principal component.

Figure 1A shows the body weights and blood glucose levels of TSOD mice. The numbers of analyzed samples are shown in Supplementary Table S1. The average body weight and average blood glucose level did not exceed the suggested thresholds for defining obesity in TSOD mice^30^ (body weight ≥40 g) and prediabetes in rodents^31^ (blood glucose level ≥200 mg/dL) until 7 weeks of age. Our results were consistent with those of previous studies reporting that the metabolic syndrome onset in TSOD mice was seen at approximately 8–12 weeks of age^20,24,27^.

In order to identify the pre-disease state before metabolic syndrome, we comprehensively assessed gene expression profiles in the adipose tissues of TSOD and control Tsumura, Suzuki, Non-Obesity (TSNO) mice^22^ at each age using DNA microarrays. A principal component analysis (PCA) was initially performed in order to reveal overall trends in transcriptome data (Fig. 1B,C). Eight meaningful principal components (PCs) were estimated (Fig. 1B). The two strains of TSOD and TSNO mice were almost completely separated in the PCA plot using PC1 and PC2 (Fig. 1C). The data points of individual mice were roughly aligned along age, except for the last 2 weeks in each group. The overall shifts to the right and down may reflect common developmental or age-related changes. No outlier was found, and thus, the quality and reliability of all gene expression data were satisfactory. In addition, no apparent abrupt change in TSOD mice was observed at every week of age in the PCA plot, which indicated the necessity of additional elaborated statistical analyses to reveal the pre-disease state before metabolic syndrome. The results of other meaningful PCs are included in Supplementary Fig. S1.

**Figure 2:**
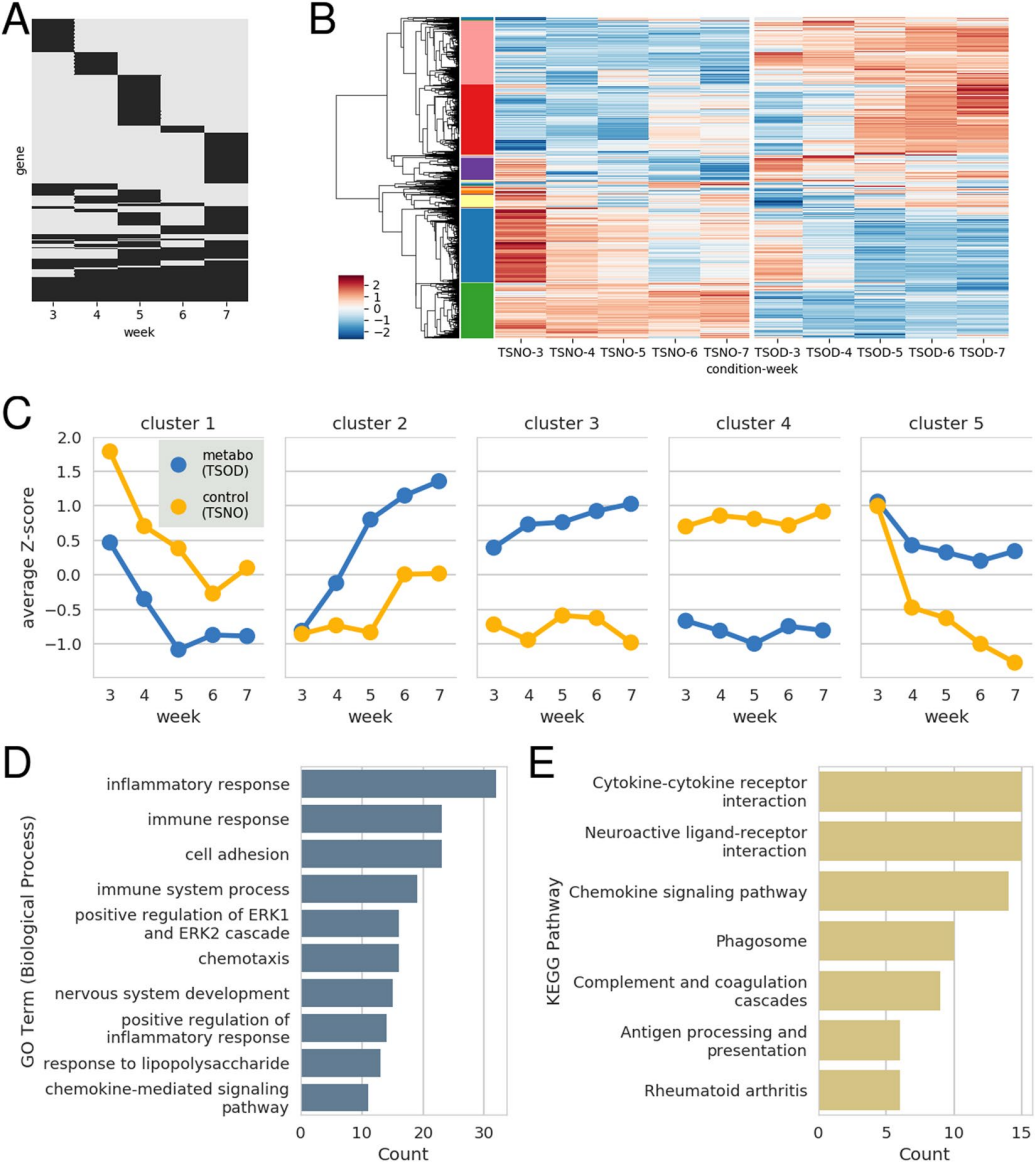
DEGs between TSOD and TSNO mice. (**A**) Overlap pattern of the five DEG sets. Each row corresponds to a gene that was included in at least one DEG set, and black regions indicate the time periods at which the gene was differentially expressed. (**B**) Heatmap of the union set of the DEGs. The color scale shows the z-score (per row) of the log expression. The left color boxes next to the dendrogram denote gene clusters obtained from hierarchical clustering. For more details, see *Clustering analysis* in Materials and Methods. (**C**) The time series of the average z-score of the top five largest clusters. (**D**) GO terms enriched in the second cluster. Each bar shows the number of genes with the corresponding term in the analyzed gene set. (**E**) KEGG pathways enriched in the second cluster. The meaning of each bar is similar to that in **D**. Regarding other cluster results of the enrichment analyses, see Supplementary Figs S2–S5.

Furthermore, differentially expressed genes (DEGs) between TSOD and TSNO mice were selected. We compared each age of the two mice groups (for example, TSOD mice (*n* = 5) versus TSNO mice (*n* = 5) at 3 weeks of age). The numbers of DEGs were 889, 852, 1321, 664, and 1296 for 3, 4, 5, 6, and 7 weeks of age, respectively. The total number of genes that were expressed differentially in at least one time period was 2665. Approximately 50% genes were included in only one DEG set, and the others were shared by two or more DEG sets (Fig. 2A). Although Venn diagrams are commonly used to visualize the overlap pattern of a few gene sets, we selected a different method because there were five DEG sets.

The union set of the DEGs was separated into clusters (Fig. 1B), and the top five largest clusters were extracted (Fig. 2C). The sizes of the five clusters were 611, 579, 530, 457, and 179. The properties of the five clusters were analyzed using the Gene Ontology (GO) enrichment analysis and Kyoto Encyclopedia of Genes and Genomes (KEGG) pathway enrichment analysis. For example, the GO (Fig. 2D) and KEGG pathway (Fig. 2E) enrichment analyses revealed that the second cluster contained many genes associated with inflammation and immune responses. Other cluster results from the enrichment analyses are included in Supplementary Figs S2–S5.

**Figure 3:**
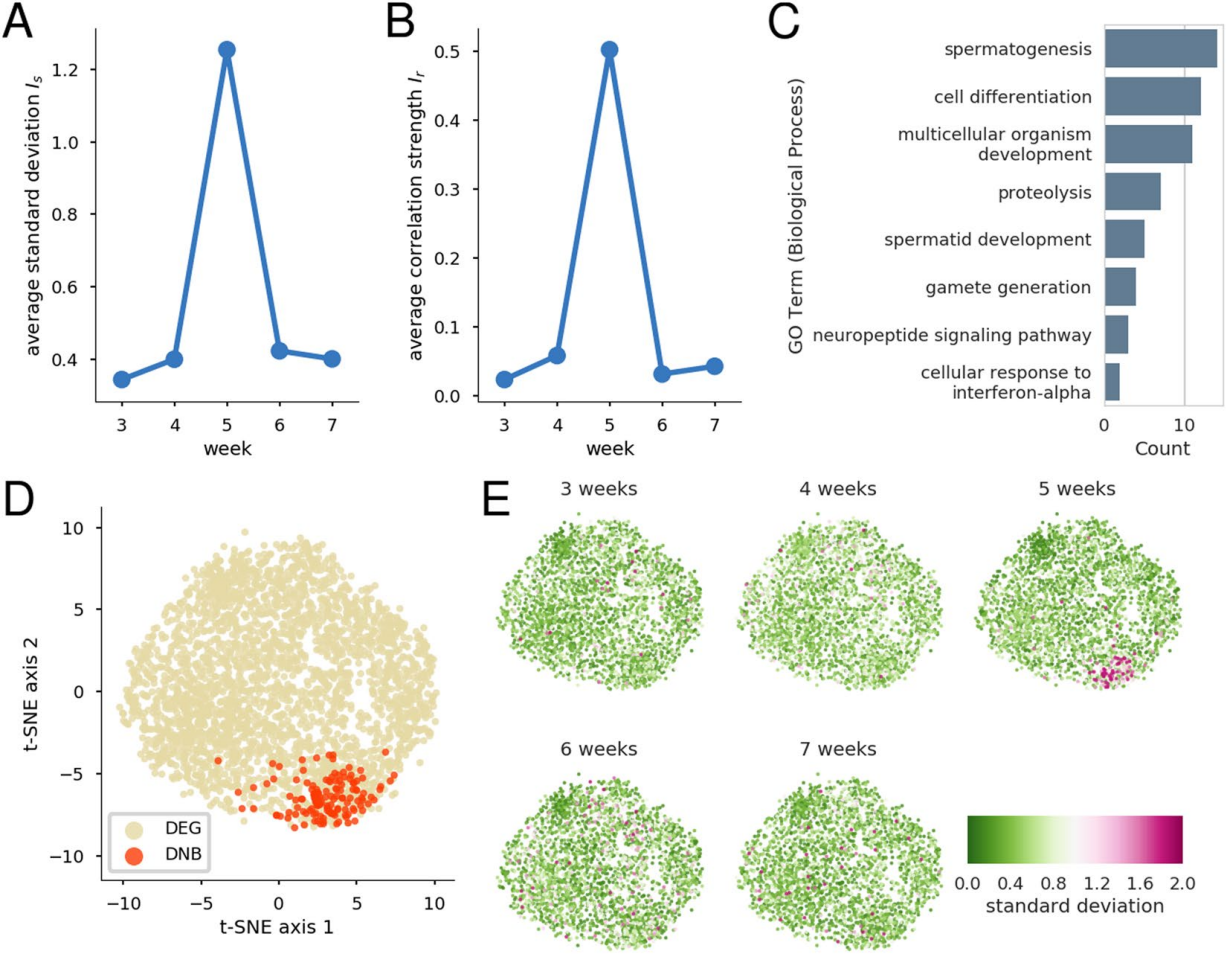
Characteristics of 147 DNB genes. (**A**) The average standard deviation *I*_*s*_. (**B**) The average correlation strength *I*_*r*_. (**C**) Enriched GO annotations. No KEGG pathway was enriched in the DNB genes. (**D**) A t-SNE plot of the union set of the DNB genes and 2665 DEGs. The overlapping genes were colored as DNB genes. For more details, see *Dimensionality reduction* in Materials and Methods. (**E**) Spatio-temporal fluctuation patterns of the DNB genes and DEGs. The color scale shows the standard deviation.

We then searched DNB genes using a simplified version of the original method^5^ (see *DNB selection* in Materials and Methods) and found a group of 147 genes (Table 1) that showed a marked peak at 5 weeks of age in TSOD mice in the average standard deviation *I*_*s*_ (Fig. 3A) and average correlation strength *I*_*r*_ (Fig. 3B). The simultaneous increases in these two statistics indicated that the DNB genes temporally showed unusually large fluctuations as well as strong correlations at the time period. The emergence of such a gene cluster has been suggested as a possible early warning signal of any abrupt change in health conditions^5^. Therefore, our results suggested that TSOD mice at 5 weeks of age, which were several weeks earlier than the onset of metabolic syndrome^20,24,27^, were in the pre-disease state preceding any essential change in the progression from a healthy state to metabolic syndrome.

**Table 1:** List of 147 DNB genes obtained from the 5-week-old TSOD mouse group. The gene symbols are sorted alphabetically.

List of 147 DNB genes					
*Actl10*	*Adam20*	*Adam4*	*Adam5*	*Adam6a*	*Als2cr11*
*Ankrd22*	*Btbd35f13*	*Capza3*	*Ccdc155*	*Ccdc54*	*Ccdc89*
*Cd55b*	*Cmtm2a*	*Cmtm2b*	*Cox7b2*	*Cox8c*	*Cst9*
*Dazl*	*Ddx4*	*Defa29*	*Dmrtb1*	*Dnajc5b*	*Eqtn*
*Fam178b*	*Fam71f2*	*Fbxw10*	*Fcrla*	*Gata3*	*Gk2*
*Gm14725*	*Gm20877*	*Gm20878*	*Gm21637*	*Gm6309*	*Gm6370*
*Gm6583*	*Gm6588*	*Gm6890*	*Gm8702*	*Got1l1*	*Grxcr2*
*Gtsf1l*	*Il31*	*Iqcf6*	*Kcnj3*	*Kcnmb4*	*Klk1b24*
*Lyzl6*	*March10*	*Mbd3l1*	*Morn2*	*Mroh8*	*Myc*
*Odf3*	*Olfr165*	*Oxct2a*	*Pcsk6*	*Pdha2*	*Pebp4*
*Pgc*	*Piwil1*	*Plcz1*	*Ppp1r2-ps7*	*Prok2*	*Prps1l1*
*Prr22*	*Prr27*	*Prss35*	*Prss37*	*Prss38*	*Prss52*
*Pth2*	*Rbakdn*	*Rbm44*	*Rnf17*	*Sf3b3*	*Sgms2*
*Sh3d21*	*Sh3gl3*	*Slc22a23*	*Slc2a3*	*Slc47a2*	*Slco6b1*
*Slx*	*Sox3*	*Sox5os3*	*Spanxn4*	*Spata3*	*Spata31d1c*
*Spata45*	*Spin2d*	*Sstr2*	*Ssty1*	*Stpg4*	*Syngr4*
*Tbc1d21*	*Tex101*	*Tex36*	*Tex48*	*Thegl*	*Tmem217*
*Tmem30c*	*Tmem35a*	*Trim69*	*Tssk2*	*Tssk6*	*Tuba3b*
*Tubd1*	*Ubqln3*	*Vmac*	*Vsig1*	*Vwa5b1*	*Wdcp*
*Zfp541*	*Zik1*	*Znrf4*	*1700006A11Rik*	*1700009C05Rik*	*1700011M02Rik*
*F17Rik*	*1700025D23Rik*	*1700026J12Rik*	*1700030L20Rik*	*1700031F10Rik*	*1700034J05Rik*
*L16Rik*	*1700061N14Rik*	*1700092M07Rik*	*1700099I09Rik*	*1700113H08Rik*	*1700126A01Rik*
*K09Rik*	*4732460I02Rik*	*4921524L21Rik*	*4930415O20Rik*	*4930449C09Rik*	*4930449I24Rik*
*I11Rik*	*4930522O17Rik*	*4930529F21Rik*	*4930544D05Rik*	*4930571K23Rik*	*4930579F01Rik*
*E13Rik*	*4933402N22Rik*	*4933406F09Rik*

We assessed the reproducibility of our DNB selection procedure using a leave-one-out approach. One sample was removed temporally from either 5-week-old TSOD mice data or 5-week-old TSNO mice data. DNB genes were then selected in the same manner, but using only the remaining samples. The genes obtained were compared with the original 147 genes. The overlap size was 74.6±12.0 (mean ± standard error of mean), suggesting an intermediate level of reproducibility of the DNB selection procedure.

Most GO annotations enriched in the DNB genes were associated with reproduction, such as spermatogenesis and spermatid development (Fig. 3C), which would not be directly related to the pathogenesis of metabolic syndrome. The KEGG pathway enrichment analysis gave no significant result. A possible reason for the failure of the GO and KEGG enrichment analyses is that these analyses are based on existing knowledge, and thus they could have difficulty for characterizing the DNB genes obtained from a novel aspect related to the pre-disease state.

In order to visualize the spatio-temporal fluctuation patterns of the DNB genes and other genes, we located the union set of the 147 DNB genes and 2665 DEGs on a two-dimensional plane using the t-distributed stochastic neighbor embedding (t-SNE)^32^ method (Fig. 3D). We selected this dimensionality reduction method because the DNB genes were mostly concentrated in one region. Figure 3E shows the spatio-temporal fluctuation patterns using the two-dimensional coordinates. Most of the DNB genes exhibited large fluctuations at 5 weeks of age in TSOD mice, whereas the majority of the other genes did not. Although a few non-DNB genes also showed large fluctuations at 5 weeks of age, they were excluded because of the lack of strong correlations with the DNB genes.

## Discussion

The present study used the DNB theory to detect the pre-disease state before metabolic syndrome (Fig. 3A,B). This extends the scope of the DNB theory in two directions. The first direction is about the variety of disorders. We previously reported that the DNB theory was able to detect the pre-disease state solely before diabetes^12^. On the other hand, metabolic syndrome is characterized by several disorders, such as hyperglycemia, hypertension, and dyslipidemia. Therefore, the present study is important in that the applicability of the DNB theory to such complex diseases has been shown.

Second, the progression of metabolic syndrome is relatively slow, and thus our results support the idea that the DNB theory is applicable to both acute and chronic diseases. The DNB theory is based on critical transitions^6^, which are abrupt changes between distinct states usually accompanied by hysteresis, namely healthy and disease states. Therefore, the DNB theory was mainly applied to data on acute diseases (for example, acute respiratory distress syndrome and influenza) to detect their pre-disease states^5,14^. On the other hand, many chronic diseases, such as metabolic syndrome, do not necessarily show apparently abrupt or discontinuous changes at the disease onset, and the health condition seems to be getting worse continuously. However, even in these cases, abrupt changes may occur in the gene expression profiles. In addition, the existence of a positive feedback loop in obesity has been suggested^33^, indicating that moving from a disease state back to a healthy state is more difficult than vice versa. This suggests a one-directional critical transition from a healthy state to a disease state, although generally the opposite-directional recovery process can be another critical transition^34^. Therefore, the DNB theory has been expected to be applicable to some chronic diseases as well as acute diseases, which has been confirmed in the present study.

We here explain in detail that TSOD mice at 5 weeks of age, at which we identified 147 DNB genes, did not develop metabolic syndrome. The onset of metabolic syndrome in TSOD mice is generally considered to be approximately 8–12 weeks^20,24,27^, at which many features common to human metabolic syndrome become apparent. For example, urinary glucose became detectable after 8 weeks of age^22^, and the enlargement of adipocytes and formation of crown-like structures with macrophage aggregation became observable in the visceral fat at approximately 12 weeks of age^20^. Diagnostic criteria for obesity in TSOD mice and prediabetes in rodents are suggested to be body weight ≥40 g and blood glucose level ≥200 mg/dL, respectively^30,31^. Clearly, in the present study, TSOD mice at 5 weeks of age did not meet these criteria (Fig. 1A). In addition, as previously reported^27,35^, transcriptions of proinflammatory cytokines TNF and Interleukin-6 were not largely increased at approximately 5 weeks of age (Supplementary Fig. S6). These genes were known to be upregulated by 11 weeks of age^23,35^. Expression of other representative metabolic genes^36^ also did not largely change (Supplementary Fig. S6). Taking all things into consideration, TSOD mice at 5 weeks of age were undoubtedly several weeks before the acquisition of major metabolic syndrome phenotypes. Therefore, we believe that the pre-disease state before metabolic syndrome was detected in TSOD mice at 5 weeks of age.

However, it is important to note that some other noticeable changes are known to occur in TSOD mice as early as 5 weeks of age. For example, the level of hydroxyoctadecadienoic acid, a biomarker of oxidative stress, was reported to be significantly higher in TSOD mice at 5 weeks of age than in age-matched TSNO mice^35^. One of the possible causes of the increase in the oxidative stress level is abnormal iron metabolism, and aberrant accumulation of iron was reported in the spleen of TSOD mice at 8 weeks of age^37^. If measured, splenic iron deposition is possibly observable at earlier ages. In addition, the intestinal microbiome was reported to be different between TSOD and TSNO mice at 5 weeks of age^38^. Further investigations from various perspectives are needed for better understanding of TSOD mice at 5 weeks of age. For example, pathological examinations^37^, which are equipped with the minute power of observations, as well as metagenome^38^ and proteome analyses will be useful in future studies.

We discuss the timing of the critical transition. We think that a critical transition occurred between 5 and 6 weeks of age, and thus *I*_*s*_ and *I*_*r*_ dropped rather than continued to rise at 6 and 7 weeks of age. This interpretation does not necessarily contradict the general notion that the onset of metabolic syndrome in TSOD mice is approximately 8–12 weeks. If a critical transition occurs mainly in adipose tissues between 5 and 6 weeks of age, it may trigger further changes in other parts of the body of mice during 6–7 weeks of age or later, and eventually the major characteristics of metabolic syndrome are acquired at approximately 8–12 weeks of age. This putative scenario is also consistent with the widely accepted view that inflammation in adipose tissues causes secretion of various chemical signals called adipokines, which affect many organs, and then metabolic syndrome is induced^1–3^. We also think that multiple important changes may occur in different parts of the body and at different weeks of age during the entire course of the progression from a healthy state to metabolic syndrome in TSOD mice.

We consider whether the usage of TSNO mice as the control group was adequate for the present study. TSNO mice has been established as the control group against TSOD mice^21,22^, and many studies on TSOD mice used TSNO mice as the control group^23–27,35,37,38^. Therefore, we compared TSOD and TSNO mice. On the other hand, the two strains were known to exhibit some differences at early ages as discussed already, and it was unknown whether TSNO mice were suitable as the control group for the study of the pre-disease state. In order to resolve this issue, we compared TSOD mice at 5 weeks of age and TSOD mice at 3 weeks of age, and confirmed that similar DNB genes were selected. The number of genes was 209, and 30 of them were shared with the original 147 DNB genes. The overlap size was significantly large (p = 9.7E-33, Fisher’s exact test). The 209 genes showed sharp peaks of *I*_*s*_ and *I*_*r*_ at 5 weeks of age (Supplementary Fig. S7A,B), which are similar to the original results (Fig. 3A,B). In addition, enriched GO annotations in the 209 genes (Supplementary Fig. S7C) were also similar to the original results (Fig. 3C). Therefore, our results of the DNB selection were not largely dependent on the choice of the control group, which justifies the validity of our main results based on the comparisons between TSOD and TSNO mice.

We also discuss possible relations between the pre-disease state before metabolic syndrome and the intestinal microbiome. Intestinal dysbiosis is generally thought to be associated with metabolic syndrome. Previous studies reported that the intestinal microbiome of TSOD mice was similar to but different from that of TSNO mice^26,38^. Our experiments also confirmed differences in intestinal microbiomes of TSOD and TSNO mice at 5 weeks of age (*Intestinal microbiome analysis* in Supplementary Information and Supplementary Fig. S8). Especially, the abundance of *Bacteroides*, which is a major genus in the phylum Bacteroidetes, decreased largely in TSOD mice at 5 weeks of age. This may be associated with the imbalance between Bacteroidetes and Firmicutes, which was reported to occur in TSOD mice at 12 weeks of age^38^ and 24 weeks of age^26^. However, it is concluded that observations of the intestinal microbiome and diabetic state were independent and that their interaction and causal relationship are still unclear^38^. The altered intestinal microbiome of TSOD mice might have influenced the gene expression patterns in adipose tissues at or before the pre-disease state. It is our future work to clarify the complex etiology and pathology of metabolic syndrome with respect to the host-guest interactions using the DNB theory.

Elevated fluctuations of the DNB genes (Fig. 3) should be interpreted carefully. According to previous studies^5,12^, we calculated the DNB scores using measurements from different individuals and did not use repeated measurements from the same individual. This is because, in order to investigate the gene expression profiles in adipose tissues, a mouse was dissected for each measurement. Therefore, what we investigated were population fluctuations, a part of which would be explained by random effects (variability caused by individual-specific mean values). In order to overcome this limitation, we plan to develop a new statistical method to apply the DNB theory to peripheral blood data in clinical study.

We here discuss the time interval of the *I*_*s*_ and *I*_*r*_ signals shown in Fig. 3A,B. These statistics are expected to increase as the system’s stability decreases^5^. This means that if the system’s stability gradually decreases for a certain time interval before a critical transition, these statistics may show an increasing trend during that time interval. It is important to note that actual values fluctuate to some extent due to noise^6,8^. Although the time resolution was not very high, the two statistics appeared to begin to increase at 4 weeks of age, which is consistent with expectations. Additional measurements between 4 and 5 weeks of age are needed as further evidence. If the increasing trend is confirmed, we will be more convinced that the observed sharp peak at 5 weeks of age was caused by a decrease in the system’s stability rather than other reasons, such as temporal changes in noise intensity.

Another viewpoint is the usefulness of the *I*_*s*_ and *I*_*r*_ signals as early warning signals. If a certain signal is detectable in a short time interval only, frequent measurements are generally required to avoid missing it. Our results suggest that the expressions of the DNB genes in TSOD mice need to be assessed at least once every week to avoid missing sharp increases in *I*_*s*_ and *I*_*r*_. Therefore, even if similar early warning signals exist for humans, weekly measurements of gene expressions in individuals before metabolic syndrome are needed, which may be impractical due to the high associated costs. In order to resolve this issue, the development of cost-effective biomarkers of the pre-disease state, which are easy to measure and show signals for a longer time interval, will be needed. As discussed already, we plan to develop such new biomarkers in future research.

We here discuss the relationship between our results and critical slowing down. Critical slowing down before a critical transition is a phenomenon that recovery from perturbation becomes increasingly slow as the system’s stability decreases. We could not measure the recovery rate directly in this study because unrealizable repetitive measurements at short intervals from the same individual is generally required. On the other hand, the relative recovery rate can be estimated because the recovery rate is known to be approximately and inversely proportional to the largest eigenvalue of the sample covariance matrix of the state variables^19^. By using the 147 DNB genes, we estimated the relative recovery rate for each week, and observed that the estimated relative recovery rate was smallest at 5 weeks of age (Supplementary Fig. S9). This suggests critical slowing down at 5 weeks of age in TSOD mice.

Finally, we discuss how our results will contribute to mitigate the prevalence of metabolic syndrome. Diet and exercise therapies are generally the first choice for prevention and treatment of metabolic syndrome, but their adherence is often poor among individuals with obesity. Pharmacotherapy is used when diet and exercise therapies alone are not sufficient, but most medicines are targeting each symptom in metabolic syndrome, such as hyperglycemia, which can lead to polypharmacy. On the other hand, we demonstrated the existence of a notable pre-disease state before metabolic syndrome defined by characteristic behavior of the DNB genes (Fig. 3). This suggests the possibility to design novel and effective therapeutic strategies for preventing metabolic syndrome, enabling just-in-time preemptive interventions.

## Materials and Methods

### Spontaneous mouse model of metabolic syndrome

Three-week-old male TSOD (Tsumura, Suzuki, Obese, Diabetes) mice and TSNO (Tsumura, Suzuki, Non-Obesity) mice were purchased from the Institute for Animal Reproduction (Ibaraki, Japan). Mice were housed in groups of two or three per cage, maintained at 24±2 °C on a 12-hour light and 12-hour dark cycle, and given normal chow diet (MF; Oriental Yeast Co., Ltd., Tokyo, Japan) and water ad libitum. Nonfasting blood glucose concentrations in tail vein blood and body weights were measured in 3-, 4-, 5-, 6-, and 7-week-old TSOD and TSNO mice. After measurements, the mice in each group were dissected to collect epididymal white adipose tissue under anesthesia to minimize suffering. Regarding each adipose tissue sample of individual mice, the total RNA was extracted using the RNeasy Total RNA Extraction kit (Qiagen, Valencia, CA, USA). The numbers of samples taken for subsequent analyses are shown in Supplementary Table S1. This animal study was performed in strict accordance with the recommendations in the Guide for the Care and Use of Laboratory Animals of University of Toyama. The protocol was approved by the Committee on the Ethics of Animal Experiments of the University of Toyama.

### Microarray assay

In order to investigate the gene expression profiles of each mouse, the Agilent SurePrint G3 Mouse Gene Expression 8 × 60 K Microarray Kit (Agilent Technologies, Santa Clara, CA, USA) was used. Cyanine-3 (Cy3)-labeled cRNA was prepared from 0.1 μg total RNA using the Low Input Quick Amp Labeling Kit (Agilent) according to the manufacturer’s instructions, followed by RNeasy column purification (QIAGEN, Valencia, CA). Dye incorporation and cRNA yield were checked with the NanoDrop ND-2000 Spectrophotometer (Thermo Fisher Scientific, Waltham, MA, USA) and Agilent 2100 Bioanalyzer (Agilent). A total of 0.6 μg of Cy3-labeled cRNA was fragmented at 60 °C for 30 minutes in a reaction volume of 25 μl containing 1 × Agilent fragmentation buffer and 2 × Agilent blocking agent following the manufacturer’s instructions. On completion of the fragmentation reaction, 25 μl of 2 × Agilent hybridization buffer was added to the fragmentation mixture and hybridized to SurePrint G3 Mouse Gene Expression 8 × 60 K Microarray (Agilent) at 65 °C for 17 hours in a rotating Agilent hybridization oven. After hybridization, microarrays were washed at room temperature for 1 minute with GE Wash Buffer 1 (Agilent) and at 37 °C for 1 minute with GE Wash buffer 2 (Agilent). Slides were scanned immediately after washing on the Agilent G2505C Microarray Scanner System (Agilent) using the one color scan setting for 8 × 60 k array slides (scan area 61 mm × 21.6 mm, scan resolution 3 μm, the dye channel was set to green, and photomultiplier tube (PMT) gain was set to 100 percent). Scanned images were analyzed with Feature Extraction Software 12.0.3.1 (Agilent) using default parameters to obtain background-subtracted and spatially detrended Processed Signal intensities.

### Microarray data preprocessing

The probe names in the raw dataset were converted to gene symbols according to the latest annotation table available at https://earray.chem.agilent.com/earray/catalogGeneLists.do?action=displaylist. Probe names without any gene symbol annotation were removed. When multiple probe names were assigned to a single gene symbol, the mean value was taken. Gene expression values were then divided by the 2% trimmed mean (the mean value calculated by discarding the lowest 2% and highest 2% values) in each sample in order to normalize the dataset. Normalized values were base-2 log-transformed.

### Dimensionality reduction

Two dimensionality reduction methods were used in the present study: the principal component analysis (PCA) for the all gene expression data and t-distributed stochastic neighbor embedding (t-SNE)^32^ for visualizing results of the DNB analysis. PCA is based on an eigendecomposition of a sample covariance matrix, or equivalently, a singular value decomposition of a mean-centered data matrix^19^. The number of meaningful principal components (PCs) were determined by a variant of parallel analysis. 100 matrices with the same size as the original data were generated by shuffling the original data. PCA was then performed for each randomized matrix to calculate 95 percentiles of the eigenvalues. The number of the original eigenvalues above them was taken as the meaningful PCs. We also used t-SNE in order for nonlinear transformation of high-dimensional data points to a two-dimensional plane. It is important to note that both axes are equally relevant, and the measure for the mapping quality of t-SNE, which is of the form of Kullback-Leibler divergence, is invariant under rotations or reflections of the plane. We used a t-SNE implementation in the scikit-learn package of python, and set the perplexity to 100 and multiplier for early exaggeration to 10. Default values were used for the other parameters. The dissimilarity between genes was calculated as 1 − |*r*_*ij*_| + |*r*′_*ij*_|, where *r*_*ij*_ and *r*′_*ij*_ are the correlation coefficients between the *i*th and *j*th genes of 5-week-old TSOD and TSNO mice, respectively.

### Extraction of differentially expressed genes

Differentially expressed genes (DEGs) are genes with expression values that markedly change between different conditions or groups. In the present study, DEGs were extracted based on fold-changes and hypothesis testing. In fold-change filtering, the arithmetic mean of the log-transformed values (or equivalently, the logarithm of the geometric mean in the original scale) was calculated for each gene in each group. Genes that exhibited more than one inter-group difference, which corresponded to more than a two-fold change in the original scale, were taken as the first group of DEG candidates. On the other hand, two-tailed Welch’s t-tests were performed for each gene using log-transformed values. In order to alleviate the large risk of Type-I errors in multiple hypothesis testing, we adjusted the significance level by the Benjamini-Hochberg (BH) procedure, which controls the supremum of the expected value of the false discovery rate (FDR). The genes for which the null hypothesis was rejected based on the adjusted significance level (E(FDR) ≤ 0.05) were taken as the second group of DEG candidates. We extracted the intersection of the two candidate groups as DEGs.

### Clustering analysis

We used a hierarchical clustering method to find gene clusters that showed similar time evolutions. Since the dynamic range of the gene expression profiles was large, we initially performed z-score normalization for each gene. Dissimilarity between genes was evaluated based on 1 − *r*_*ij*_, where *r*_*ij*_ is the correlation coefficient between the *i*th and *j*th genes. The average linkage method was then used to calculate the dendrogram. Genes were separated into clusters with a cutoff value of 0.5 for inter-cluster dissimilarity.

### Enrichment analysis

Enrichment analyses of GO annotations and the KEGG pathways were performed using the web tool of the DAVID (Database for Annotation, Visualization and Integrated Discovery) database^39^ (https://david.ncifcrf.gov). The purpose of the enrichment analysis is to characterize a set of genes, such as DEGs. The target gene set is compared with many other well-characterized gene sets (for example, genes sharing the same GO annotation or those involved in the same KEGG pathway), and overlaps are calculated. Based on this information, we may reveal what types of genes are largely included in the target gene set.

In order to establish whether an overlap between two gene sets is significantly large, the one-tailed Fisher’s exact test based on a hypergeometric distribution is generally used. Its p-value is defined as follows:

1 $$p=\sum _{k=x}^{min({n}_{1},{n}_{2})}\frac{(\begin{array}{c}N-{n}_{1}\\ {n}_{2}-k\end{array})(\begin{array}{c}{n}_{1}\\ k\end{array})}{(\begin{array}{c}N\\ {n}_{2}\end{array})}=1-\sum _{k=0}^{x-1}\,\frac{(\begin{array}{c}N-{n}_{1}\\ {n}_{2}-k\end{array})(\begin{array}{c}{n}_{1}\\ k\end{array})}{(\begin{array}{c}N\\ {n}_{2}\end{array})},$$

where *N* is the total number of genes of the target organism, *n*_1_ is the size of the gene set under analysis, *n*_2_ is the size of another gene set whose characteristics are known, and *x* is the overlap between the two gene sets. It is important to note that the p-values provided by the DAVID’s web tool are based on a modified version of Fisher’s exact test, in which *x* and *n*_1_ are substituted by *x* − 1 and *n*_1_ − 1, respectively. These modifications make the test more conservative and suppress false positives. Although multiple hypothesis testing was involved, we initially picked up all gene sets with *p* ≤ 0.05 and *x* ≥ 2 for exploratory purposes. Top 10 gene sets were then selected based on the size of overlaps.

### DNB selection

DNB genes are genes with expression values that exhibit unusually large fluctuations in a collective manner at the pre-disease state. Since no standardized method for DNB selection has been established currently, various approaches have been investigated^5,14,16^. In the present study, we used a simplified version of the original method^5^.

We here introduce some notations. Let *X* = {*x*_*ik*_}(*i* = 1, …, *N*, *k* = 1, …, *K*) denote a gene expression dataset restricted to a certain condition (for example, 5-week-old TSOD mice), where *N* is the total number of genes and *K* is the number of samples in the subdata. The gene-wise mean *m*_*i*_(*X*), standard deviation *s*_*i*_(*X*), and correlation coefficient *r*_*ij*_(*X*) are defined as follows:

2 $${m}_{i}(X)=\frac{{\sum }_{k=1}^{K}\,{x}_{ik}}{K},\,(i=1,\ldots ,N),$$

3 $${s}_{i}(X)=\sqrt{\frac{{\sum }_{k=1}^{K}\,{({x}_{ik}-{m}_{i}(X))}^{2}}{K-1}},\,(i=1,\ldots ,N),$$

4 $${r}_{ij}(X)=\frac{{\sum }_{k=1}^{K}\,({x}_{ik}-{m}_{i}(X))({x}_{jk}-{m}_{j}(X))}{(K-1){s}_{i}(X)\,{s}_{j}(X)},\,(i,j=1,\ldots ,N).$$

It is important to note that only some of the correlation coefficients were actually calculated in the following procedure. Let *Y* = {*y*_*ik*_}(*i* = 1, …, *N*, *k* = 1, …, *L*) denote the data of the control group, where *L* is the number of samples in *Y*. The gene-wise mean *m*_*i*_(*Y*), standard deviation *s*_*i*_(*Y*), and correlation coefficient *r*_*ij*_(*Y*) are similarly defined.

**Figure 4:**
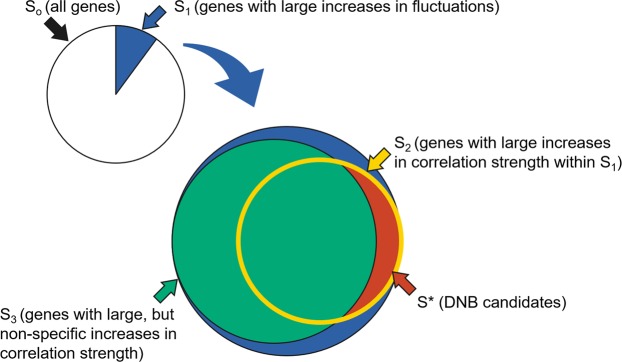
Schematic illustration of DNB selection. *S*_0_: indices of all genes. *S*_1_: indices of genes with large increases in fluctuations, which are selected from *S*_0_. *S*_2_: indices of genes with large increases in correlation strength within *S*_1_, which are selected from *S*_1_. *S*_3_: indices of genes with large, but non-specific increases in correlation strength against a broad range of genes, which are selected from *S*_1_. S^*^: indices of DNB candidates with large increases in correlation strength specifically within *S*_1_, each element of which is in *S*_2_ but not in *S*_3_.

Based on the statistics described above, we selected candidate genes of DNB in three steps (Fig. 4). First, genes that showed relatively large increases in fluctuations were selected based on the ratio of the standard deviations:

5 $${v}_{i}^{(1)}=\frac{{s}_{i}(X)}{{s}_{i}(Y)},\,(i=1,\ldots ,N).$$

The indices corresponding to the highest ${\theta }_{1}$ (%) values of ${v}_{i}^{(1)}$ were selected and denoted as ${S}_{1}$. The selection of a parameter value for ${\theta }_{1}$ as well as other parameters is explained later.

Second, a cluster of genes that temporally showed strong correlations was extracted based on the average change of the strength of the correlation coefficients:

6 $${v}_{i}^{(2)}=\sum _{j\in {S}_{1}}\,(|{r}_{ij}(X)|-|{r}_{ij}(Y)|),\,(i\in {S}_{1}).$$

The indices corresponding to the highest *θ*_2_ (%) values of ${v}_{i}^{(2)}$ were extracted and denoted as *S*_2_. We did not use here a clustering method, such as hierarchical clustering, for three reasons: (1) ${v}_{i}^{(2)}$ is easy to calculate, (2) only one parameter is involved, and (3) a selection step among multiple clusters is not necessary.

Third, genes that showed non-specific increases in the correlation strength against a broad range of genes were excluded based on the following statistics:

7 $${v}_{i}^{(3)}=\sum _{j\in {S}_{0}\backslash {S}_{1}}\,(|{r}_{ij}(X)|-|{r}_{ij}(Y)|),\,(i\in {S}_{1}),$$

where ${S}_{0}=\{1,\ldots ,N\}$ is the set of all gene indices. The indices corresponding to the highest ${\theta }_{3}$ (%) values of ${v}_{i}^{(3)}$ were extracted and denoted as ${S}_{3}$. The genes corresponding to ${S}^{\ast }={S}_{2}\backslash {S}_{3}$, which showed large increases in correlation strength specifically within ${S}_{1}$, were then selected as candidate genes of DNB.

We investigated various parameter values of *θ*_1_, *θ*_2_, and *θ*_3_, and obtained many DNB candidate sets. In order to evaluate the performance of each candidate set as an indicator of the pre-disease state, we calculated the average standard deviation *I*_s_ and the average correlation strength *I*_r_ as follows:

8 $${I}_{{\rm{s}}}=\frac{1}{|{S}^{\ast }|}\sum _{i\in {S}^{\ast }}\,{s}_{i}(Z),$$

9 $${I}_{{\rm{r}}}=\frac{2}{|{S}^{\ast }|(|{S}^{\ast }|-1)}\sum _{i,j\in {S}^{\ast },\,i < j}\,|{r}_{ij}(Z)|-c,$$

where S^*^ is the indices of a DNB candidate set, |S^*^| denotes the cardinality of S^*^, *Z* = {*z*_*ik*_}(*i* = 1, …, *N*, *k* = 1, …, *M*) is a subdata of a certain condition, and *c* is a correction term that depends on the sample size (*c* = 0.50 for 4 samples and *c* = 0.42 for 5 samples). The correction term represents the expected correlation strength of two independent random variables both following the standard normal distribution. We introduced this term because a systematic increase in correlation strength was observed in subdata with only 4 samples. Based on the two DNB scores *I*_*s*_ and *I*_*r*_, we searched a gene set that showed a sharp peak both in *I*_*s*_ and *I*_*r*_ at a particular time period, and found a gene set consisting of 147 genes. The corresponding parameter values were *θ*_1_ = 10%, *θ*_2_ = 50%, and *θ*_3_ = 80%.

Although the original method^5^ also proposed the third statistic, the average correlation strength between the DNB variables and the others, we did not use it because the third statistic frequently does not behave as expected. Theoretically, the third statistic works only when the dominant eigenvector of the Jacobian matrix of a dynamical system contains exactly zero elements^5^, and even a small deviation from zero may result in wrong behavior. In fact, the third statistic for the 147 DNB genes was almost constant (Supplementary Fig. S10). A possible reason is that the dominant eigenvector of the Jacobian matrix in this case did not contain exactly zero elements. This may occur if we regard the elements of the eigenvector as continuous random variables following certain continuous distributions because the conditions of real living organisms are perpetually fluctuating. In that case, the probability measure of any one of the elements being exactly zero becomes zero. Therefore, all elements take non-zero values almost surely as long as the assumptions described above hold true.

## Supplementary information

Supplementary Information

## Data Availability

Source codes used in the present study for statistical analyses will be made available in the GitHub repository under MIT license (https://github.com/okumakito/dnb-tsod). The microarray datasets generated and analyzed during the present study are available in the Gene Expression Omnibus (GEO) repository through GEO series accession number GSE112653 (https://www.ncbi.nlm.nih.gov/geo/query/acc.cgi?acc=GSE112653).
