# Supplementary material for: Identifying pre-disease signals before metabolic syndrome in mice by dynamical network biomarkers
Source: Sci Rep. 2019 Jun 24;9:8767. doi: 10.1038/s41598-019-45119-w (PMC6591167; doi:10.1038/s41598-019-45119-w)
Supplement: Supplementary file 1 — Supplementary Information [file 41598_2019_45119_MOESM1_ESM.pdf]

Supplementary Information—Identifying pre-disease signals before metabolic syndrome in mice by dynamical network biomarkers

Keiichi Koizumi, Makito Oku, Shusaku Hayashi, Akiko Inujima, Naotoshi Shibahara, Luonan Chen, Yoshiko Igarashi, Kazuyuki Tobe, Shigeru Saito, Makoto Kadowaki, Kazuyuki Aihara

Intestinal microbiome analysis

Intestinal microbiome analysis of fecal specimens from TSOD and TSNO mice at 3, 4, and 5 weeks of age was conducted by TechnoSuruga Laboratory (Shizuoka, Japan) with the terminal restriction fragment length polymorphism (T-RFLP) method (Supplementary Fig. S8). DNA was extracted from the frozen fecal specimens according to [1], and its purity and concentration were measured using NanoDrop ND8000 (Thermo Fisher Scientific, USA). Bacterial 16S ribosomal DNA sequences were then amplified using polymerase chain reaction according to [2] and [3] with 6-carboxyfluorescein (6-FAM) labelled primers 516F and 1510R. The resulting amplicons were digested with FastDigest BseLI (BslI) restriction enzyme (Thermo Fisher Scientific, USA) at 37 °C for 10 min. Fragment analysis was then performed using ABI PRISM 3130xl Genetic Analyzer (Applied Biosystems, USA). Detected peaks were assigned to operational taxonomic units (OTUs). Multiple peaks within a certain range were assigned to the same OTU according to [2] and [3]. The OTUs were then annotated with predicted bacterial groups. Bacteria belonging to the class Clostridia were divided into clusters according to [4]. OTUs annotated with zero or more than one predicted bacterial groups were categorized as others.

References

1. Takahashi, S. *et al.* Development of a prokaryotic universal primer for simultaneous analysis of *Bacteria* and *Archaea* using next-generation sequencing. *PLOS ONE* **9**, e105592, DOI: https://doi.org/10.1371/journal.pone.0105592 (2014).

2. Nagashima, K. *et al.* Application of new primer-enzyme combinations to terminal restriction fragment length polymorphism profiling of bacterial populations in human feces. *Appl. Environ. Microbiol.* **69**, 1251–1262, DOI: https://doi.org/10.1128/AEM.69.2.1251-1262.2003 (2003).

3. Nagashima, K. *et al.* Phylogenetic analysis of 16S ribosomal RNA gene sequences from human fecal microbiota and improved utility of terminal restriction fragment length polymorphism profiling. *Biosci. Microflora* **25**, 99–107, DOI: https://doi.org/10.12938/bifidus.25.99 (2006).

4. Collins, M. D. *et al.* The phylogeny of the genus *Clostridium*: Proposal of five new genera and eleven new species combinations. *Int. J. Syst. Bacteriol.* **44**, 812–826, DOI: https://dx.doi.org/10.1099/00207713-44-4-812 (1994).

Supplementary Table S1. The numbers of analyzed samples. Samples with insufficient quality for microarray assay were excluded.

	Week				
Group	3	4	5	6	7
TSNO	5	5	5	5	5
TSOD	5	5	5	4	5

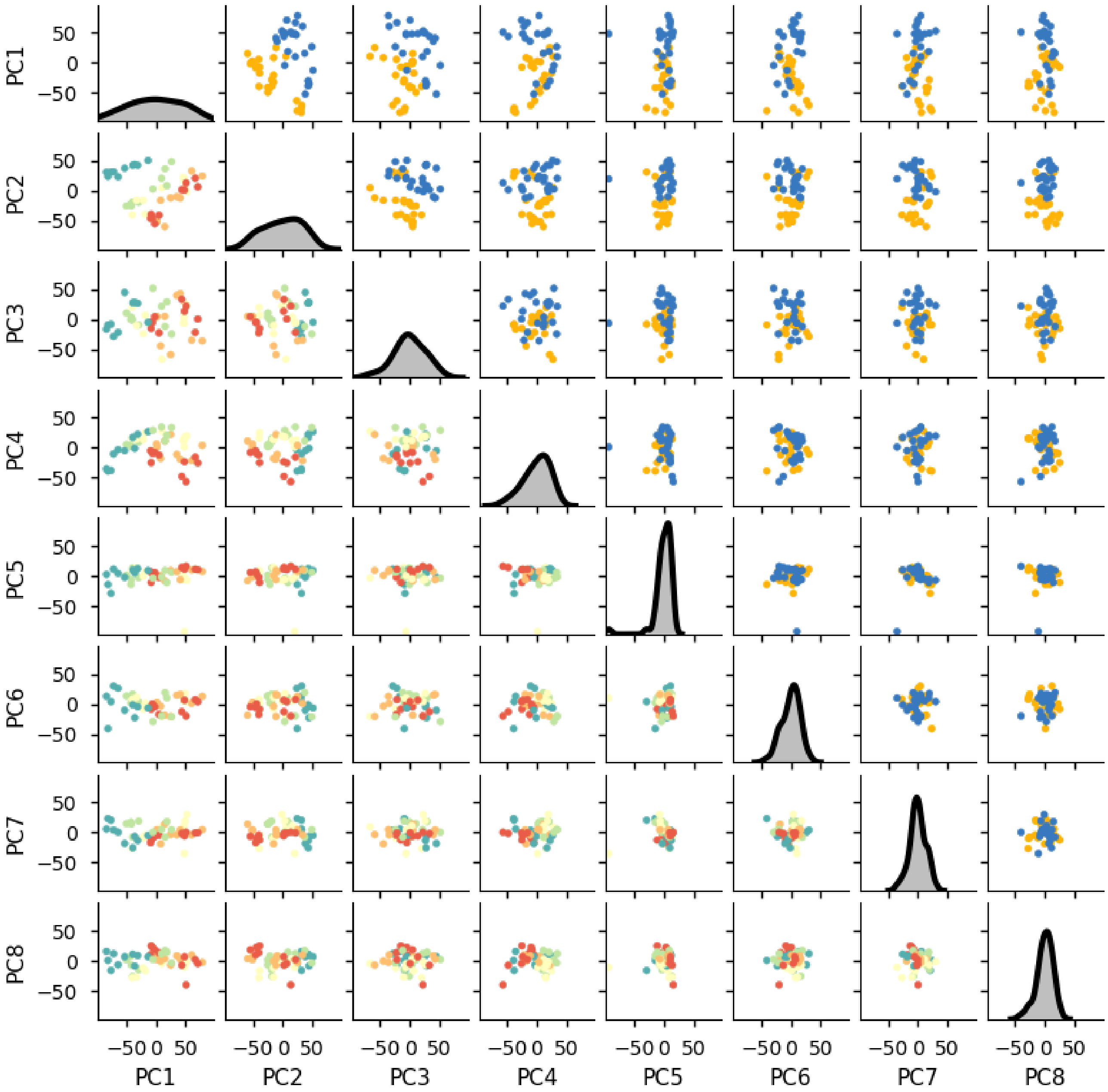

Supplementary Figure S1. PCA plots of all meaningful components. Colors in the upper off-diagonal plots represent strains: TSOD mice (blue) and TSNO mice (yellow). Colors in the lower off-diagonal plots represent age of mice. The color assignment is the same to that in Fig. 1C. Diagonal plots show distributions. The explained variance ratios are 25.6 %, 13.4 %, 9.2 %, 6.5 %, 3.6 %, 3.0 %, 2.5 %, and 2.5 % for PC1 to PC8.

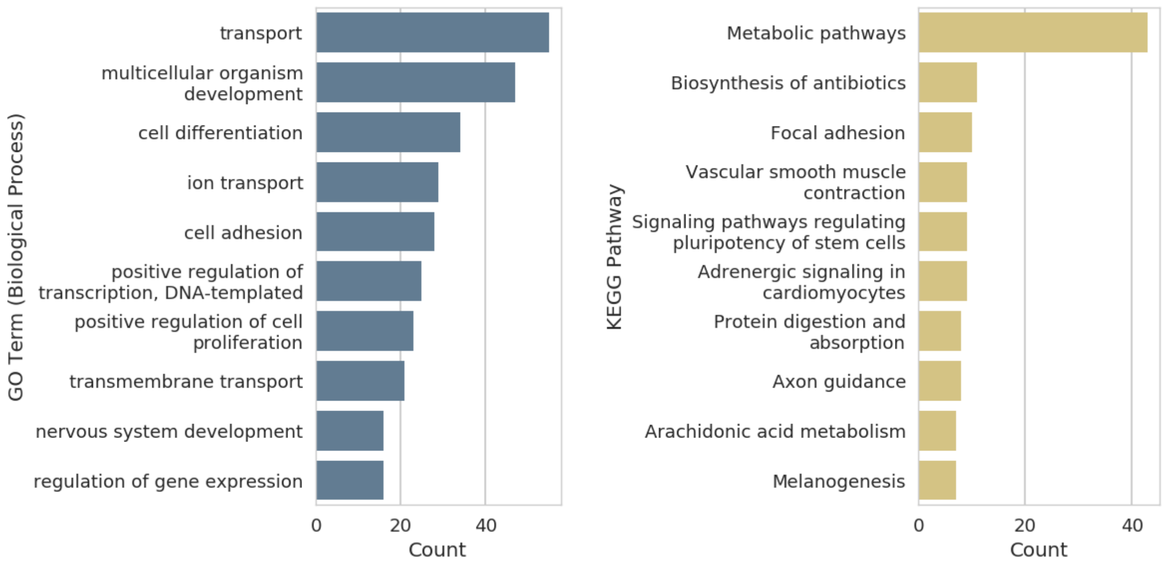

Supplementary Figure S2. GO terms and KEGG pathways enriched in the first cluster.

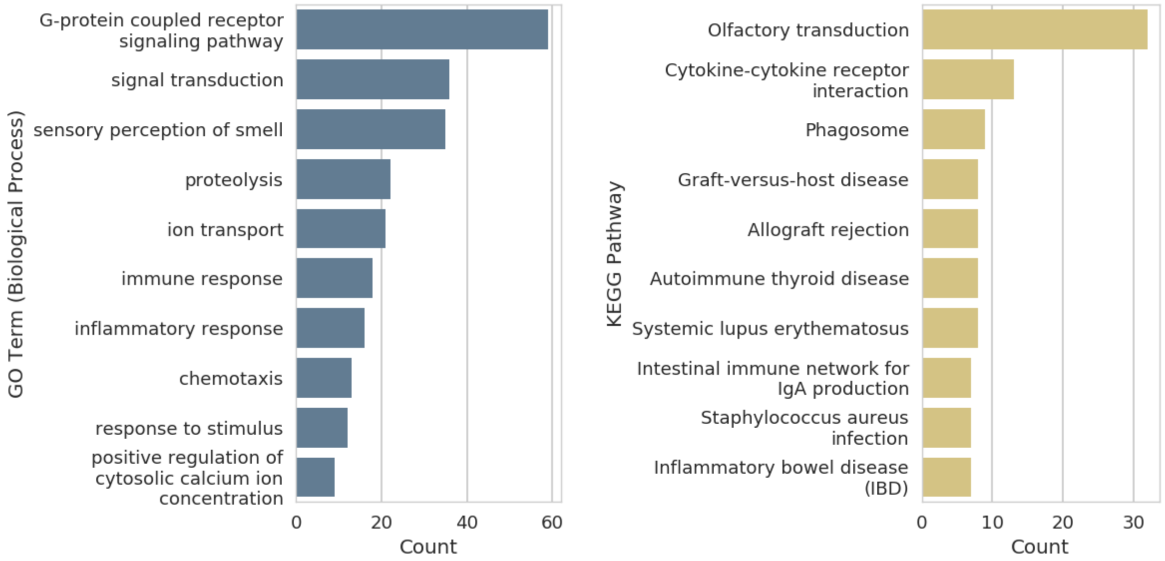

Supplementary Figure S3. GO terms and KEGG pathways enriched in the third cluster.

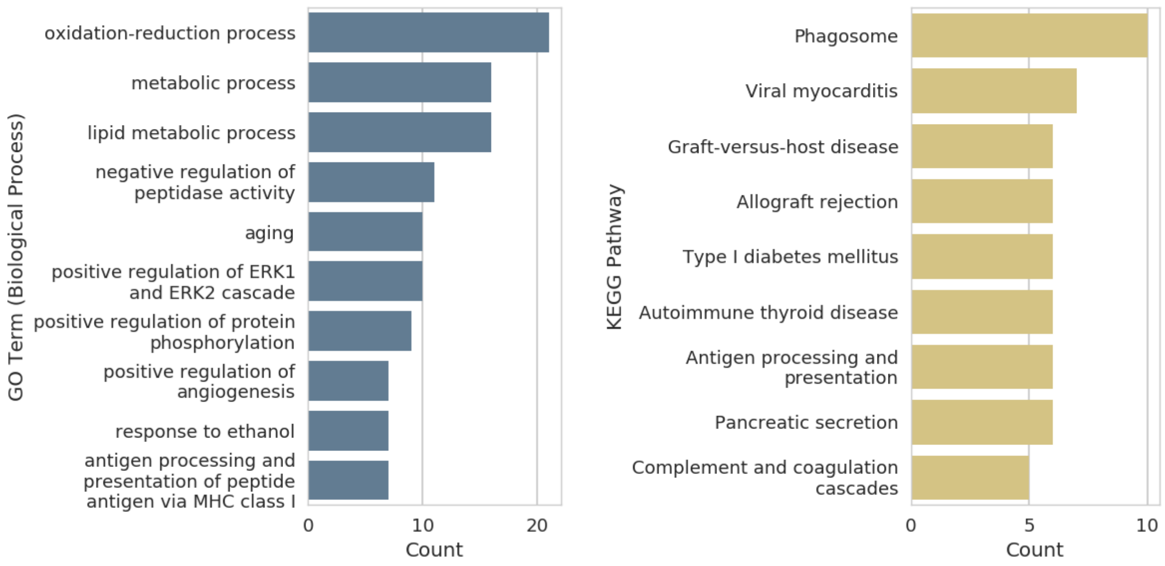

Supplementary Figure S4. GO terms and KEGG pathways enriched in the fourth cluster.

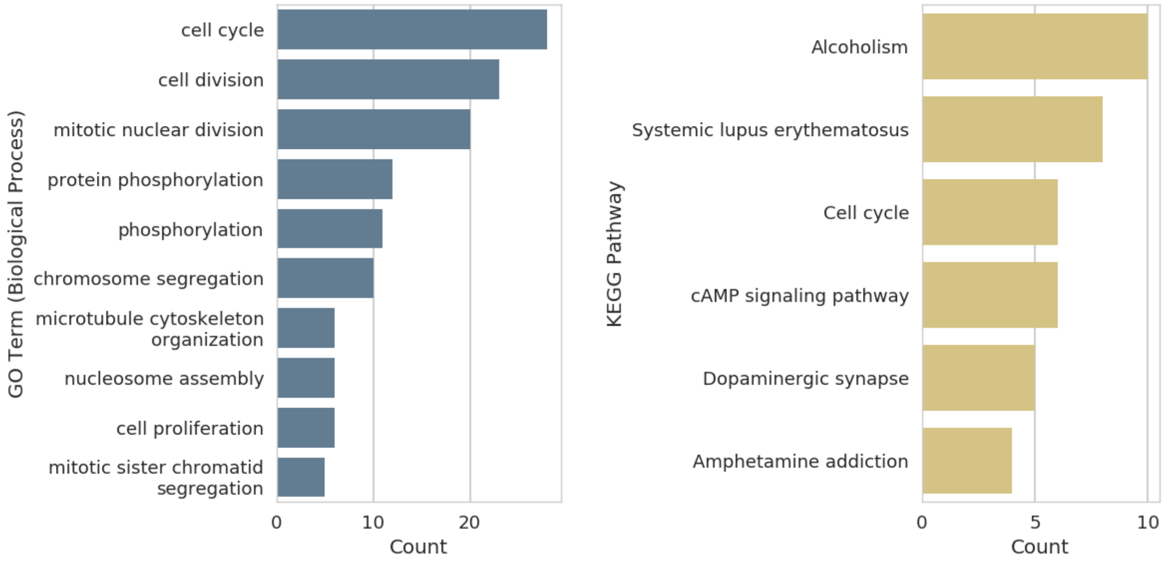

Supplementary Figure S5. GO terms and KEGG pathways enriched in the fifth cluster.

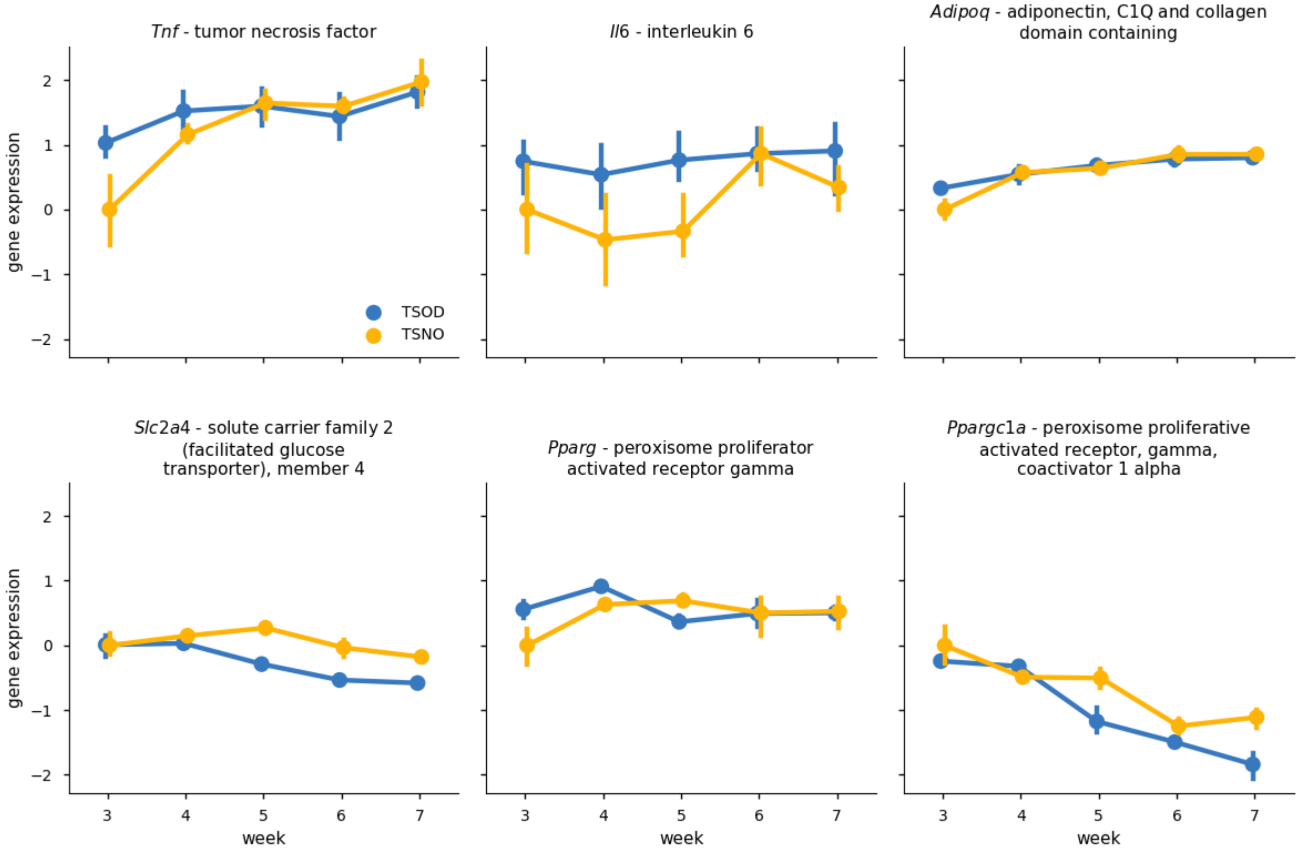

Supplementary Figure S6. Log expression of pro-inflammatory cytokines (*Tnf* and *Il6*) and metabolic genes (*Adipoq*, *Slc2a4*, *Pparg*, and *Ppargc1a*). The mean values of 3-week-old TSNO mice were subtracted in each plot. Error bars show 95 % confidence intervals. The values of *Il6* expression were not reliable because they were close to the background noise level.

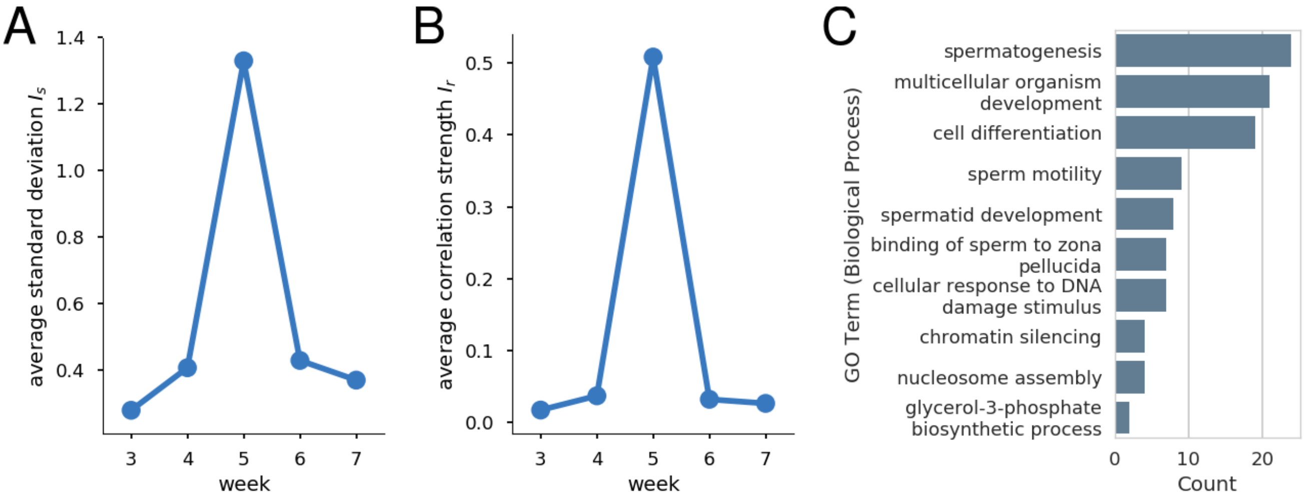

Supplementary Figure S7. Characteristics of 209 DNB genes obtained from the comparison between TSOD mice at 5 weeks of age and TSOD mice at 3 weeks of age ($\theta_1 = 5$ %, $\theta_2 = 35$ %, and $\theta_3 = 30$ %). (**A**) The average standard deviation I_s. (**B**) The average correlation strength I_r. (**C**) Enriched GO annotations.

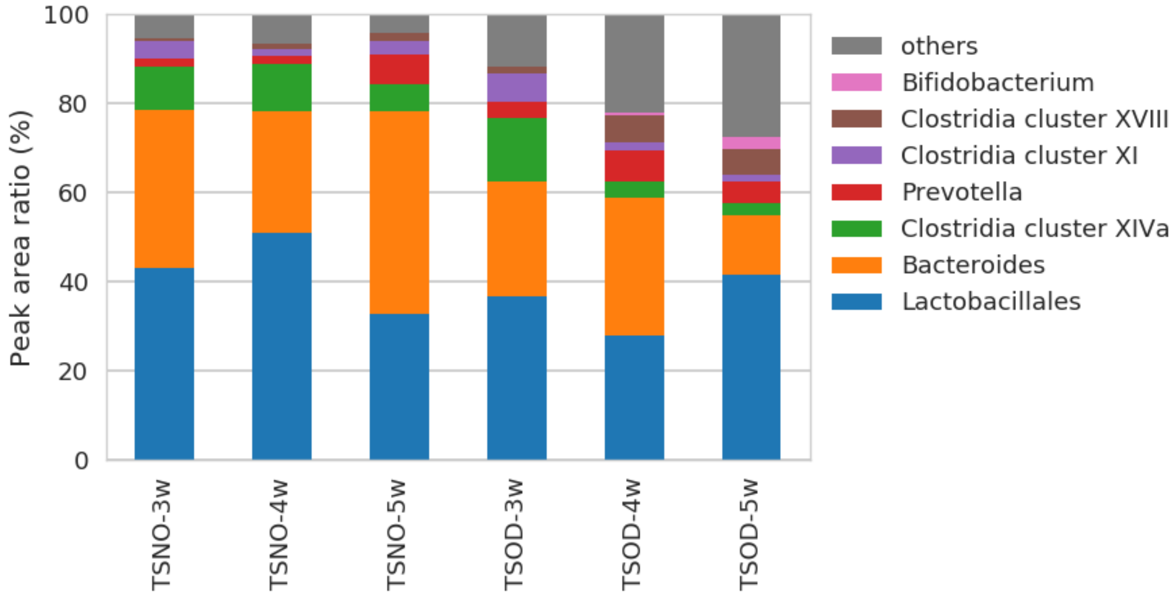

Supplementary Figure S8. Intestinal microbiomes of TSOD and TSNO mice at 3, 4, and 5 weeks of age. The peak area ratios of predicted bacterial groups measured using the T-RFLP method are shown.

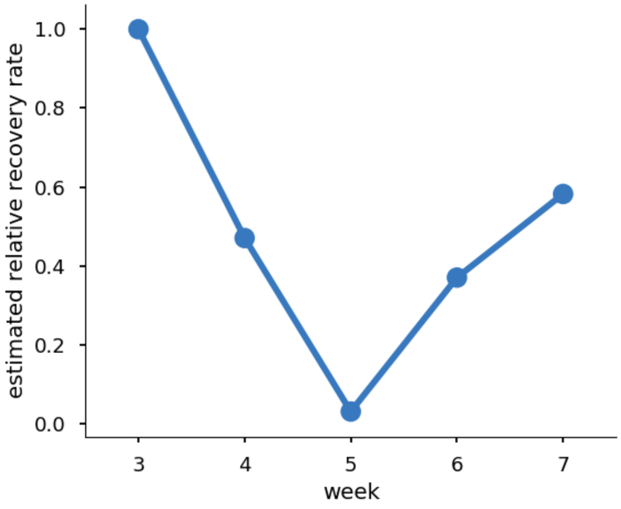

Supplementary Figure S9. The estimated relative recovery rate. Smaller values indicate slower recovery from perturbation. The values are normalized so that the maximum value is 1.

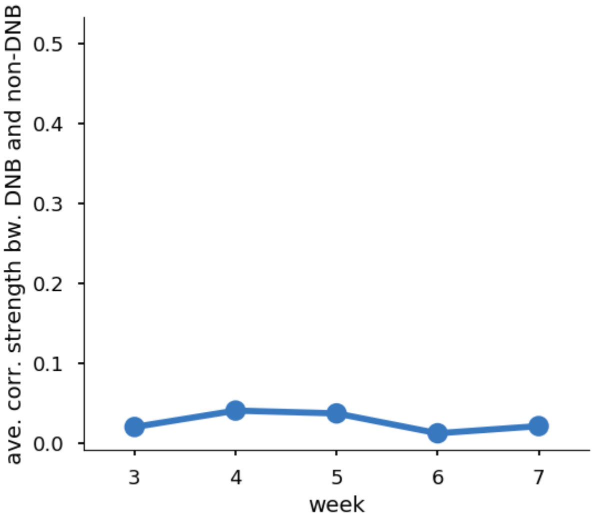

Supplementary Figure S10. The average correlation strength between 147 DNB genes and the others. The range of the vertical axis is the same to that of Fig. 3B. The sample-size correction term was used, as in I_r.
